# Cyclooxygenase-2 Signalling Pathway in the Cortex is Involved in the Pathophysiological Mechanisms in the Rat Model of Depression

**DOI:** 10.1038/s41598-017-00609-7

**Published:** 2017-03-28

**Authors:** Qi Chen, Ying Luo, Shengnan Kuang, Yang Yang, Xiaoyan Tian, Jie Ma, Shaoshan Mai, Lai Xue, Junqing Yang

**Affiliations:** Department of Pharmacology, Chongqing Medical University, the Key Laboratory of Biochemistry and Molecular Pharmacology, Chongqing, 400016 China

## Abstract

This study was designed to investigate the effect of the cortical cyclooxygenase-2 (COX2) pathway on depressive behaviour in rats. Meloxicam, COX2 overexpressed lentivirus and COX2 RNAi lentivirus were administered to Sprague-Dawley rats subjected to chronic unpredictable mild stress (CUMS). Behaviour tests, biochemistry and immunohistochemistry methods, enzyme-linked immunosorbent assays, western blotting and reverse transcription polymerase chain reactions were used to evaluate the changes in rat behaviour and the cortical COX2 pathway. CUMS rats showed depressive–like behaviours. The superoxide dismutase activity and cyclic adenosine monophosphate (cAMP) contents were significantly decreased, the contents of malondialdehyde, prostaglandin E2 (PGE2) and inflammatory cytokines were significantly increased. The expressions of protein kinase A (PKA) and cAMP response element-binding protein (CREB) were decreased, and the levels of brain-derived neurotrophic factor (BDNF) and COX2 were significantly increased. Meloxicam and COX2 RNAi lentivirus significantly alleviated the abnormalities induced by CUMS, while COX2 overexpressed lentivirus aggravated these abnormalities. Our results indicated that the cortical COX2 pathway was activated in CUMS rats. Inhibition of COX2 activity/expression can obviously improve depressive behaviours in CUMS rats. Upregulating COX2 expression can increase the susceptibility of rats to CUMS. An imbalance in the cortical COX2-PGE2-cAMP/PKA-CREB-BDNF signalling pathway participates in the pathogenic mechanism of depression.

## Introduction

Depression is a common mood disorder of mental illness, with the main clinical characteristics of abnormal behaviours and low spirits. This disease may cause harmful effects or even damage to the nervous, endocrine and digestive systems. According to a survey and forecast from the World Health Organization (WHO), depression will become the main cause of non-fatal diseases and will become the second-most disabling disease after cardiovascular disease by 2020^1^. As scientific research has progressed, the role of inflammation in the pathogenesis of depression has been gradually taken into consideration^2^. In a meta-analysis, *Howren MB* discovered that the levels of inflammation factors, such as C-reactive protein (CRP), interleukin-1β (IL-1β) and interleukin-6 (IL-6), in depressive patients were significantly higher than those in patients without depression and that the depressive patients often showed depressive symptoms after being given inflammatory cytokines, such as interferon-α^3^. Moreover, antidepressants can significantly improve the depressive behaviour caused by inflammation^4^. Therefore, some scholars have noted that depression could be considered a kind of chronic inflammatory disease.

Cyclooxygenase-2 (COX2) is the key enzyme for the production of a series of inflammatory cytokines. Under the stimulation of inflammatory cytokines and mitogen, COX2 can catalyse arachidonic acid (AA) to generate the corresponding prostaglandins (PGs) (PGD_2_, PGF_2α_, PGI_2_, TXA_2_ and PGE_2_), which exert different biological effects through each corresponding G protein-coupled receptor (DP, FP, IP, TP and EP) and further cause inflammation and tissue damage in the central nervous system in a PG-dependent manner^5^. Among the PGs, PGE2 is the most active. By binding to different PGE2 receptors (EP1, EP2, EP3 and EP4), it can regulate the generation of cyclic adenosine monophosphate (cAMP), increase the intracellular calcium ion concentration, activate phosphatidylinositol 3-kinase, participate in the regulation and release of nerve growth factors in the brain, promote the production of inflammatory cytokines and induce the activity of nitric oxide synthase, which mediates the brain’s nerve toxicity or protection^6–8^. There is a close relationship between the cAMP transduction pathway and depression. The cAMP pathway is involved in neuron survival and the maintenance of synaptic plasticity, and it is a common target for certain antidepressants^9^. Thus, the increase in COX2 expression affects not only the inflammatory response in the central nervous system by regulating activities of the cAMP system by activating Eps but also the neural plasticity. However, this needs to be further confirmed by comprehensive studies.

Studies have found that the expression of COX2 in the brain of rats increases significantly with the appearance of stress-caused depressive symptoms and that celecoxib, a COX2 selective inhibitor, can greatly relieve depressive behaviours^10^. Our previous study also showed that chronic unpredictable mild stress (CUMS) causes significant depressive behaviour in rats and that the oral administration of meloxicam, a COX2 inhibitor, can relieve depressive symptoms, alleviate damage of hippocampal nerve cells and up-regulate the expression of neural plasticity-related factors, such as synaptophysin (SYP), brain-derived neurotrophic factor (BDNF) post-synaptic density 95 (PSD-95) and 5-hydroxytryptamine(5-HT)1A receptor, in the hippocampus^11^. These results together suggest that the hippocampal COX2 pathway may be a target for the development of therapeutic drugs for depression.

In addition to the hippocampus, the structure and function of the cortex are also closely related with the occurrence of depression. As early as 1997, researchers reported that patients with temporal lobe injury showed a significant decrease in cortical 5-HT receptors and obvious depressive symptoms^12^. Recent neuroimaging studies have shown that gray matter volume decreases in the frontal lobe, parietal lobe, temporal lobe and many others in the cortex of first-episode depressive patients, and that the micro-structure of nerve fibres in these regions may also be destroyed^13^. Once a drug therapy was administered to increase the number of dendritic spines and repair the function of the first layer V pyramidal neurons in the medial prefrontal cortex, the depressive behaviours were lessened^14^. One study also showed that cortical COX2 activity and PGE2 levels significantly increased in the cortex and hippocampus of rats with depression^15^. As a summary, the structural and functional changes in cerebral cortical neurons are closely associated with depression. However, there has been no systemic research regarding the relationship between the COX2 pathway in the cortex and depression. It is worth studying the pathogenesis of depression through observing the changes in the COX2 pathway.

Therefore, on the basis of our previous studies, a CUMS-induced depression model was used to observe changes in the COX2 pathway in the cortex of depressive rats. The rats were then treated with a COX2 inhibitor, COX2 RNAi lentivirus and COX2 over-expressed lentivirus to observe changes in depressive behaviour and in the COX2-PGE2-cAMP/PKA-CREB-BDNF pathway in the cortex. The experimental results in the present study will contribute to understanding the relationship between the rat cortical COX2 pathway, as well as the pathophysiological mechanisms of depression.

## Results

### Effects of meloxicam on the changes in depressive-like behaviours in CUMS rats

**Figure 1:**
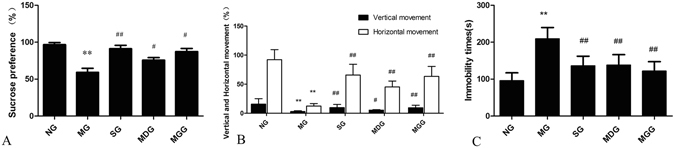
Effects of meloxicam on the changes in depressive-like behaviours in CUMS rats. (**A**) The sucrose preference (%); (**B**) The vertical and horizontal movements (%); (**C**) The immobility time(s); NG: normal group; MG: CUMS group; SG: Sertraline group; MDG: Meloxicam (1 mg.kg^−1^) group; MGG: Meloxicam (3 mg.kg^−1^). Group data shows the changes in the behaviour in rats. Data are expressed as the mean ± SD in six separate experiments. Compared with those in the normal group, the sucrose preference and the vertical and horizontal movements of rats in the open-field test were decreased, but the immobility time was increased in the CUMS group. Compared with those in the model group, the administration of sertraline and meloxicam significantly improved the sucrose preference, increased the horizontal and vertical scores and decreased the immobility time in the forced swimming test of depressive rats. **P < 0.01 vs the normal group. ^#^P < 0.05 and ^##^P < 0.01 vs the CUMS group, respectively.

Compared with that in the normal group, the sucrose preference and the vertical and horizontal movements in open-field test of CUMS rats were significantly decreased (P < 0.01) (Fig. 1A and B), whereas the immobility time in the forced swimming test was significantly increased (P < 0.01) (Fig. 1C). However, compared with that in the model group, the administration of sertraline and meloxicam improved the sucrose preference of depressive rats (P < 0.05), increased the horizontal and vertical scores in the open-field test and significantly shortened the immobility time in the forced swimming test (P < 0.01), especially in the group of rats administered meloxicam 3 mg.kg^−1^ (Fig. 1)

### Effects of meloxicam on the changes in the superoxide dismutase (SOD) activity and the contents of cAMP, malondialdehyde (MDA), PGE2, cAMP, tumour necrosis factor–α (TNF-α) and IL-1β in CUMS rat cortices

**Figure 2:**
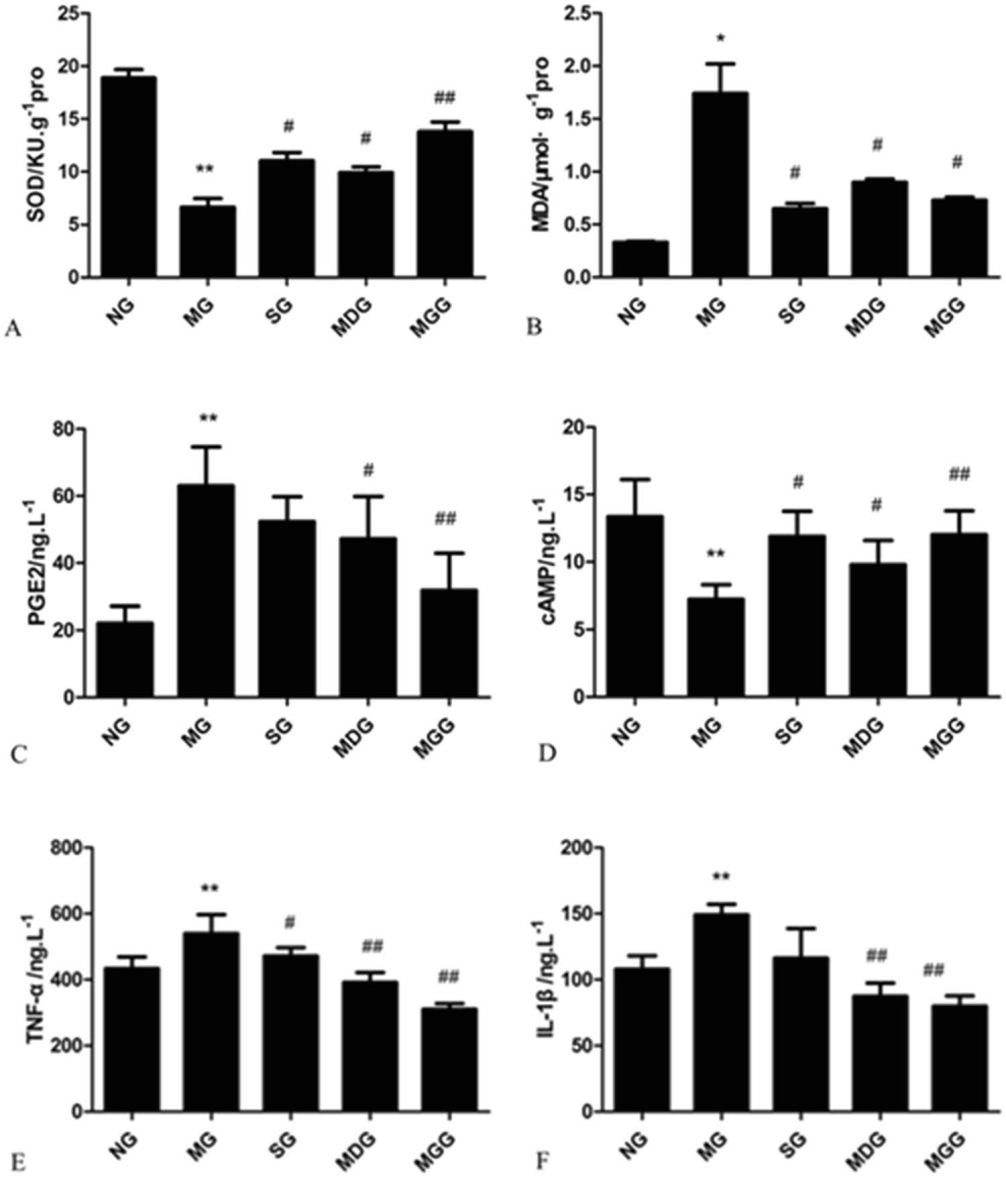
Effects of meloxicam on the changes in SOD activity and the levels of MDA, PGE2, cAMP, TNF-α and IL-1β in CUMS rat cortices. (**A**) The activity of SOD. (**B**) The content of MDA. (**C**) The content of PGE2. (**D**) The concentration of cAMP. (**E**) The content of TNF-α. (**F**) The content of IL-1β. NG: normal group. MG: CUMS group. SG: Sertraline group. MDG: Meloxicam (1 mg.kg^−1^) group. MGG: Meloxicam (3 mg.kg^−1^) group. Group data shows the changes in SOD activity and the levels of MDA, PGE2, cAMP, TNF-α and IL-1β in CUMS rat cortices. Data are expressed as the mean ± SD in six separate experiments. Compared with the normal group, the activity of SOD and the concentration of cAMP in the CUMS rats significantly decreased, while the contents of MDA, PGE2, TNF-α and IL-1β significantly increased. Compared with those in the CUMS group, the administration of sertraline and meloxicam significantly increased the activity of SOD and the concentration of cAMP, and significantly decreased the contents of MDA, PGE2, TNF-α and IL-1β. *P < 0.05 and **P < 0.01 vs the normal group, respectively. ^#^P < 0.05 and ^##^P < 0.01 vs the CUMS group, respectively.

The SOD activity of rat cortices in the model group was significantly decreased compared with that of rats in the normal group (P < 0.01), while the MDA content was significantly increased (P < 0.05). Compared with those in the model group, the administration of sertraline and meloxicam blunted the changes in SOD activity and MDA content in rat cortices (Fig. 2A and B).

The PGE2 content of rat cortices in the model group were significantly increased compared with that of the normal group (P < 0.01), while the concentration of cAMP was decreased (P < 0.01). Compared with those in the model group, the administration of meloxicam significantly decreased PGE2 content and increased the cAMP concentration in a dose-dependent manner (P < 0.05 and P < 0.01, respectively). The administration of sertraline increased the concentration of cAMP significantly (P < 0.05) but decreased PGE2 content without significance (Fig. 2C and D).

The TNF-α and IL-1β contents of rat cortices in the model group were significantly increased compared with those in the normal group (P < 0.01). The administration of meloxicam decreased these inflammatory cytokines, especially in the meloxicam group (P < 0.01), but significant differences were only seen in the changes in IL-1β content when sertraline was administered (P < 0.05) (Fig. 2E and F).

### Effects of meloxicam on protein kinase A (PKA)IIα reg protein expression in CUMS rat cortices

**Figure 3:**
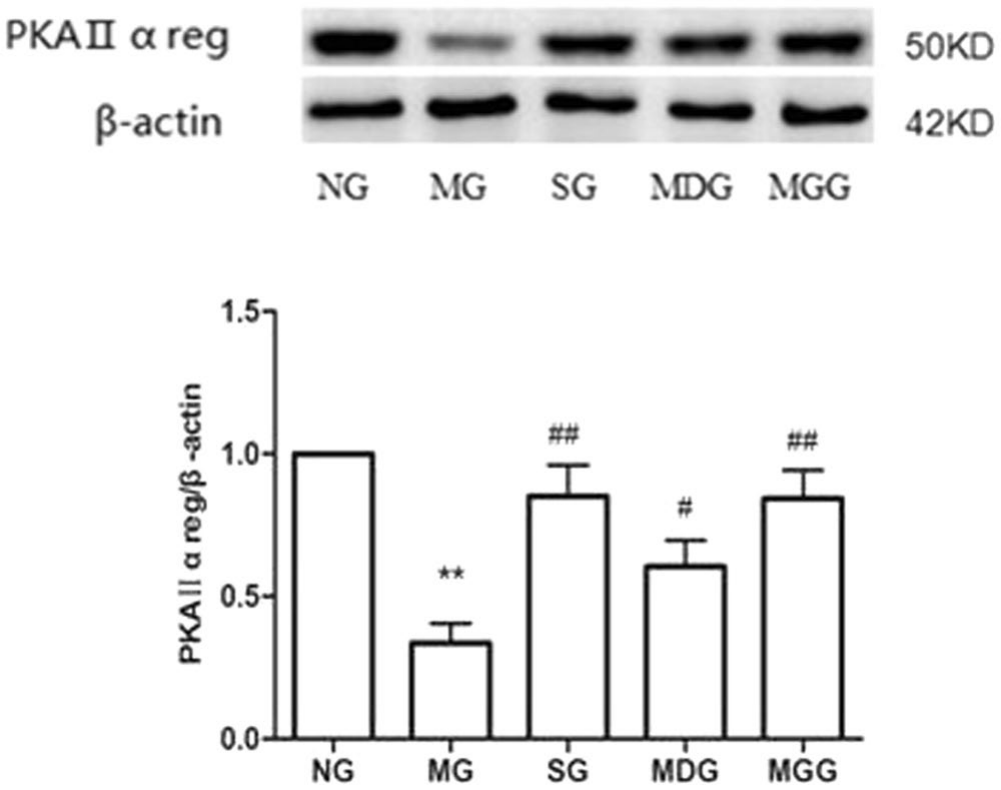
Effects of meloxicam on PKAIIα reg protein expression in CUMS rat cortices. NG: normal group. MG: CUMS group. SG: Sertraline group. MDG: Meloxicam (1 mg.kg^−1^) group. MGG: Meloxicam (3 mg.kg^−1^) group. Group data shows the changes in PKAIIα reg protein expression in CUMS rat cortices. Data are expressed as the mean ± SD in four separate experiments. PKAIIα reg protein expression significantly decreased in the CUMS group. Compared with that in the CUMS group, the administrations of sertraline and meloxicam significantly increased PKAIIα reg protein expression. **P < 0.01 compared with that in the normal group; ^#^P < 0.05 and ^##^P < 0.01 compared with that in the CUMS group, respectively.

Compared with that in the normal group, the PKAIIα reg expression in rat cortices was significantly reduced in the CUMS group (P < 0.01). The administration of sertraline increased PKAIIα reg protein expression, and the administration of meloxicam also significantly increased the PKAIIα reg protein expression in a dose-dependent manner (P < 0.05 and P < 0.01, respectively) (Fig. 3).

### Effects of meloxicam on BDNF protein expression in CUMS rat cortices

**Figure 4:**
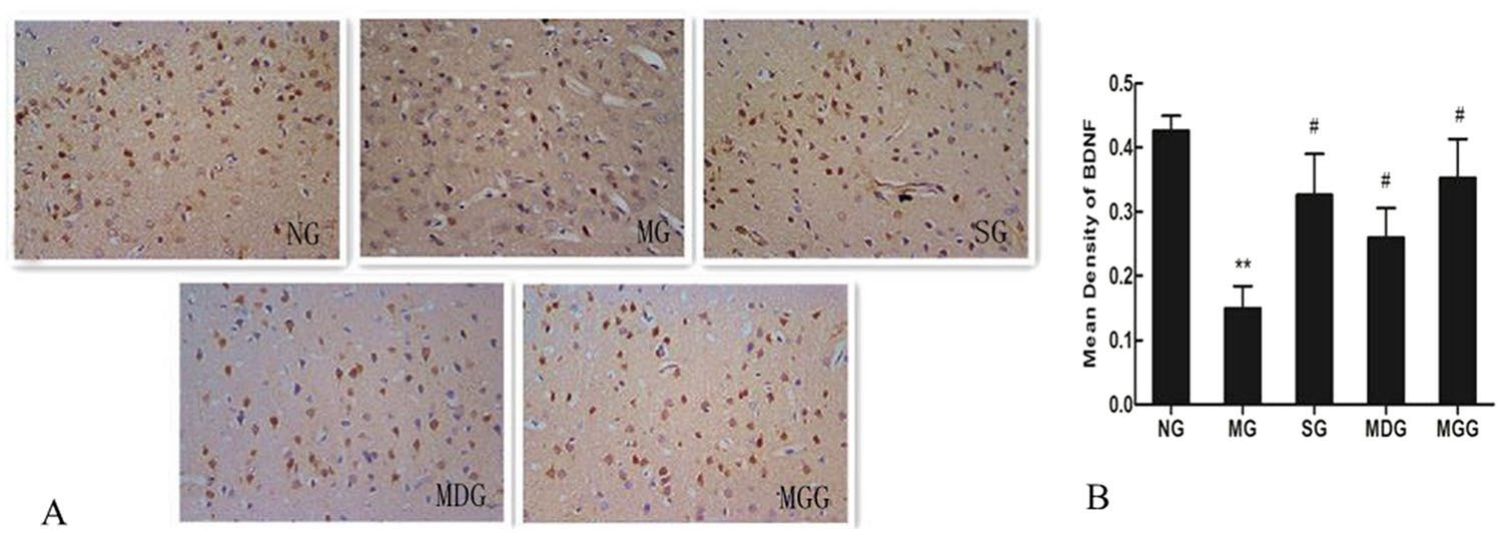
Effects of meloxicam on BDNF protein expression in CUMS rat cortices. (**A**) Changes in BDNF expression in rat cortex. (**B**) Changes in the mean density of BDNF protein expression of rat cortices. NG: normal group. MG: CUMS group. SG: Sertraline group. MDG: Meloxicam (1 mg.kg^−1^) group. MGG: Meloxicam (3 mg.kg^−1^) group. Group data shows the changes in BDNF protein expression. Data are expressed as the mean ± SD in four separate experiments. Compared with that in the control group, BDNF protein expression significantly decreased in the CUMS group. Compared with that in the CUMS group, the administrations of sertraline and meloxicam significantly increased BDNF protein expression. **P < 0.01 compared with that in the normal group. ^#^P < 0.05 and ^##^P < 0.01 compared with that in the CUMS group, respectively.

In the cortices of the normal group rats, BDNF-positive neurons were dyed deeply as brown-yellow. Compared with that of the control group, vacuolar degeneration andkaryopyknosis appeared in CUMS rat cortical neurons accompanied with less BDNF-positive cells. The administrations of sertraline and meloxicam increased BDNF-positive neurons in rat cortices (Fig. 4A). Average optical density analysis showed that BDNF-positive neurons in the model group were significantly reduced compared with those in the control group (P < 0.01). Compared with that in the model group, the average optical densities of BDNF-positive neurons in the cortices increased significantly in the sertraline- and meloxicam-treated groups (P < 0.05) (Fig. 4B).

### Effects of COX2 overexpression and RNAi on the changes in depressive-like behaviours in CUMS rats

**Figure 5:**
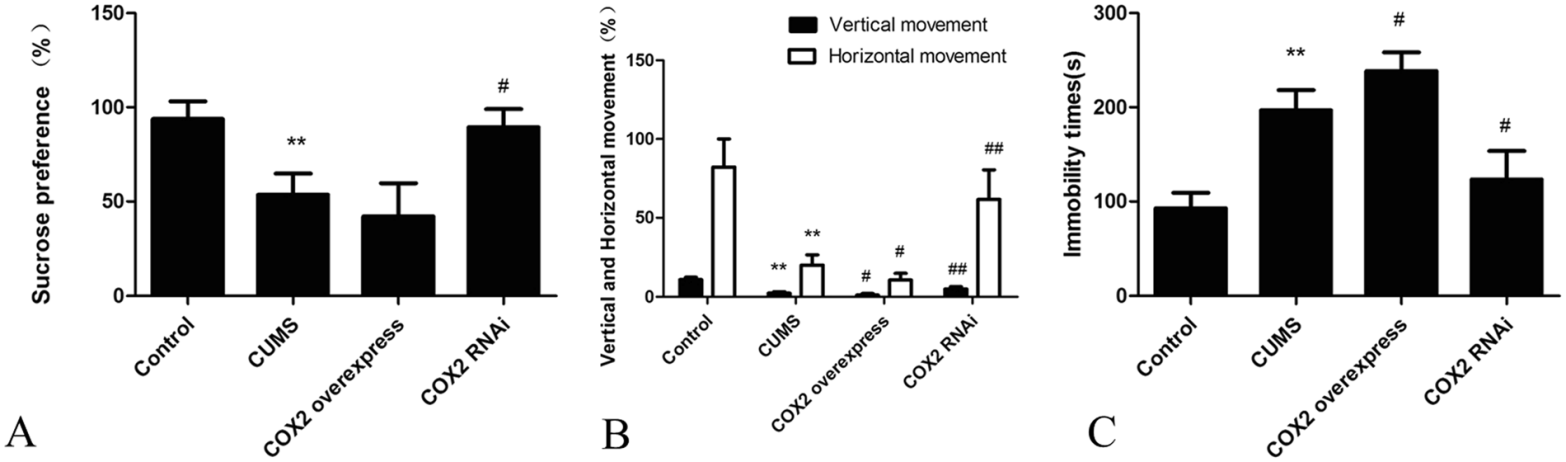
Effects of COX2 overexpression and RNAi on the changes in depressive-like behaviours in CUMS rats. (**A**) The sucrose preference (%). (**B**) The vertical and horizontal movements (%). (**C**) The immobility time(s). Group data shows the changes in the behaviour of rats. Data are expressed as the mean ± SD in six separate experiments. Compared with those in the control group, the sucrose preference, the vertical and horizontal movements of rats significantly decreased, whereas the immobility time significantly increased in the CUMS rats. Compared with that in the CUMS group, the sucrose preference and the horizontal and vertical scores were decreased, while the immobility time was significantly prolonged in the COX2 overexpressed group, but there were no significant differences in sucrose preference. In the COX2 RNAi group rats, the sucrose preference and the horizontal and vertical scores significantly increased, while the immobility time significantly decreased. **P < 0.01 vs the control group. ^#^P < 0.05 and ^##^P < 0.01 vs the CUMS group, respectively.

Compared with those in the control group, the sucrose preference and the vertical and horizontal movements in the open-field test of CUMS rats were decreased (P < 0.01), whereas the immobility time of the forced swimming test was increased (P < 0.01). Compared with that in the CUMS group, the injection of COX2 overexpressed lentivirus aggravated the depressive behaviours in the open-field tests and immobility time (P < 0.05). The changes in rat sucrose preference were not significant. However, the administration of COX2 RNAi lentivirus improved the sucrose preference of depressive rats (P < 0.05), increased their horizontal and vertical scores in the open-field test and significantly prolonged their immobility time in the forced swimming test (P < 0.01) (Fig. 5).

### Effects of COX2 overexpression and RNAi on the changes in SOD activity and the contents of cAMP, MDA, PGE2, TNF-α and IL-1β in CUMS rat cortices

**Figure 6:**
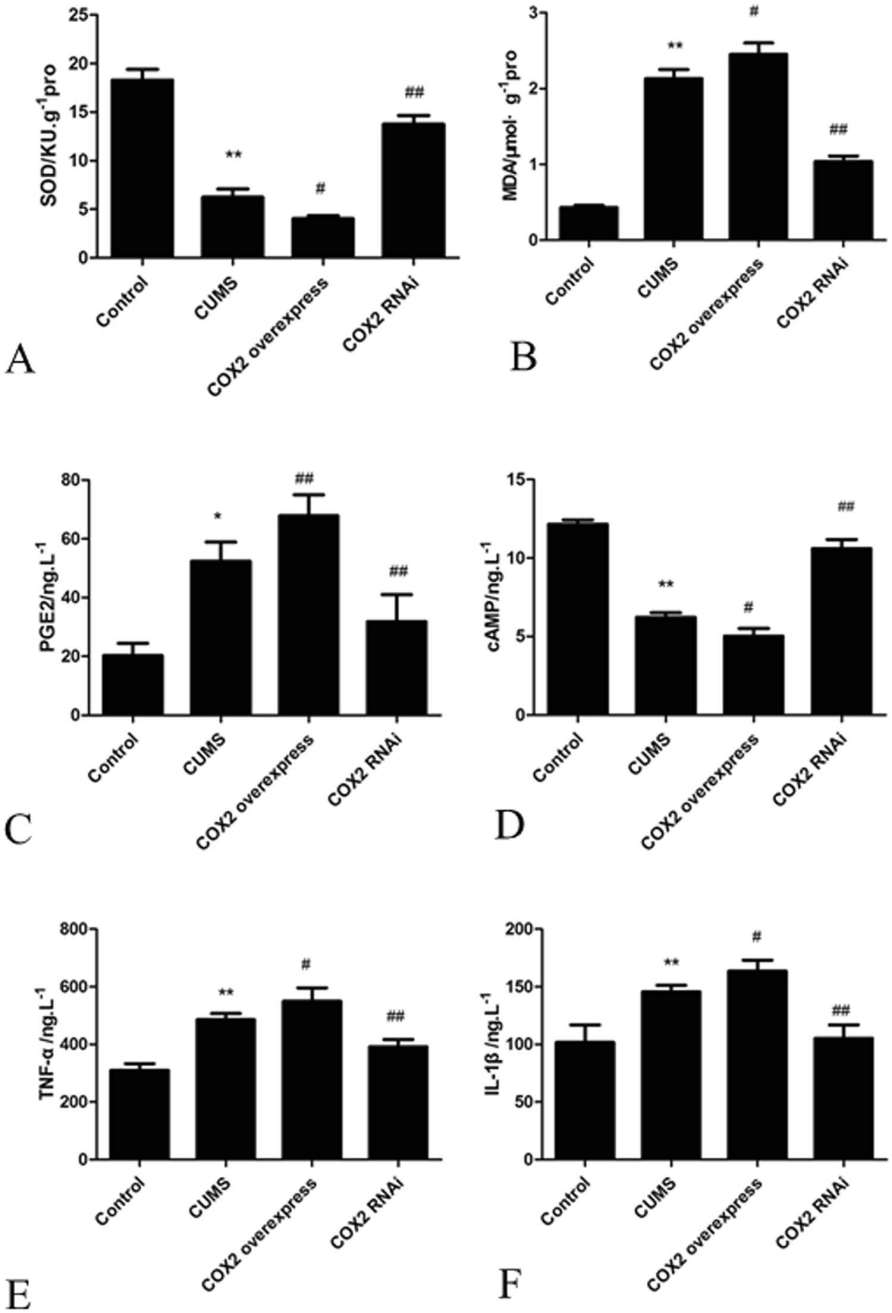
Effects of COX2 overexpression and RNAi on the changes in SOD activity and the levels of MDA, PGE2, cAMP, TNF-α and IL-1β in CUMS rat cortices. (**A**) The SOD activity. (**B**) The MDA content. (**C**) The PGE2 content. (**D**) The cAMP concentration. (**E**) The TNF-α content. (**F**) The IL-1β content. Group data shows the changes in SOD activity and the levels of MDA, PGE2, cAMP, TNF-α and IL-1β in CUMS rat cortices. Data are expressed as the mean ± SD in six separate experiments. Compared with the control group, the activity of SOD and the concentration of cAMP significantly decreased in the CUMS group, while the contents of MDA, PGE2, TNF-α and IL-1β significantly increased. Compared with those in the CUMS group, the activity of SOD and the concentration of cAMP decreased significantly, and the contents of MDA, PGE2, TNF-α and IL-1β significantly increased in the COX2 overexpressed group. The activity of SOD and the concentration of cAMP significantly increased in the COX2 RNAi group, while the contents of MDA, PGE2, TNF-α and IL-1β significantly decreased. *P < 0.05 and **P < 0.01 vs the control group, respectively. ^#^P < 0.05 and ^##^P < 0.01 vs the CUMS group, respectively.

Compared with that in the control group, the SOD activity of rat cortices in the CUMS group was significantly decreased (P < 0.01), while the MDA content was significantly increased (P < 0.05). Compared with that of the CUMS group, the injection of COX2 overexpressed lentivirus aggravated these changes (P < 0.05). However, the administration of COX2 RNAi lentivirus blunted the decrease in SOD activity and the increase in MDA content (P < 0.05) (Fig. 6A and B).

The PGE2 content of rat cortices in the CUMS group was significantly increased compared with that in the control group (P < 0.01), while the concentration of cAMP was decreased. Compared with that in the CUMS group, the injection of COX2 overexpressed lentivirus aggravated these changes (P < 0.05). However, the administration of COX2 RNAi lentivirus blunted the increase in PGE2 content and the decrease in cAMP concentration (P < 0.01) (Fig. 6C and D).

The TNF-α and IL-1β contents of rat cortices in the CUMS group were significantly increased compared with those in the control group (P < 0.01). The injection of COX2 overexpressed lentivirus further significantly increased these inflammatory cytokines (P < 0.05), while the administration of COX2 RNAi lentivirus significantly decreased these inflammatory cytokines (P < 0.01) (Fig. 6E and F).

### Effects of COX2 overexpression and RNAi on the expressions of COX2 protein and mRNA

**Figure 7:**
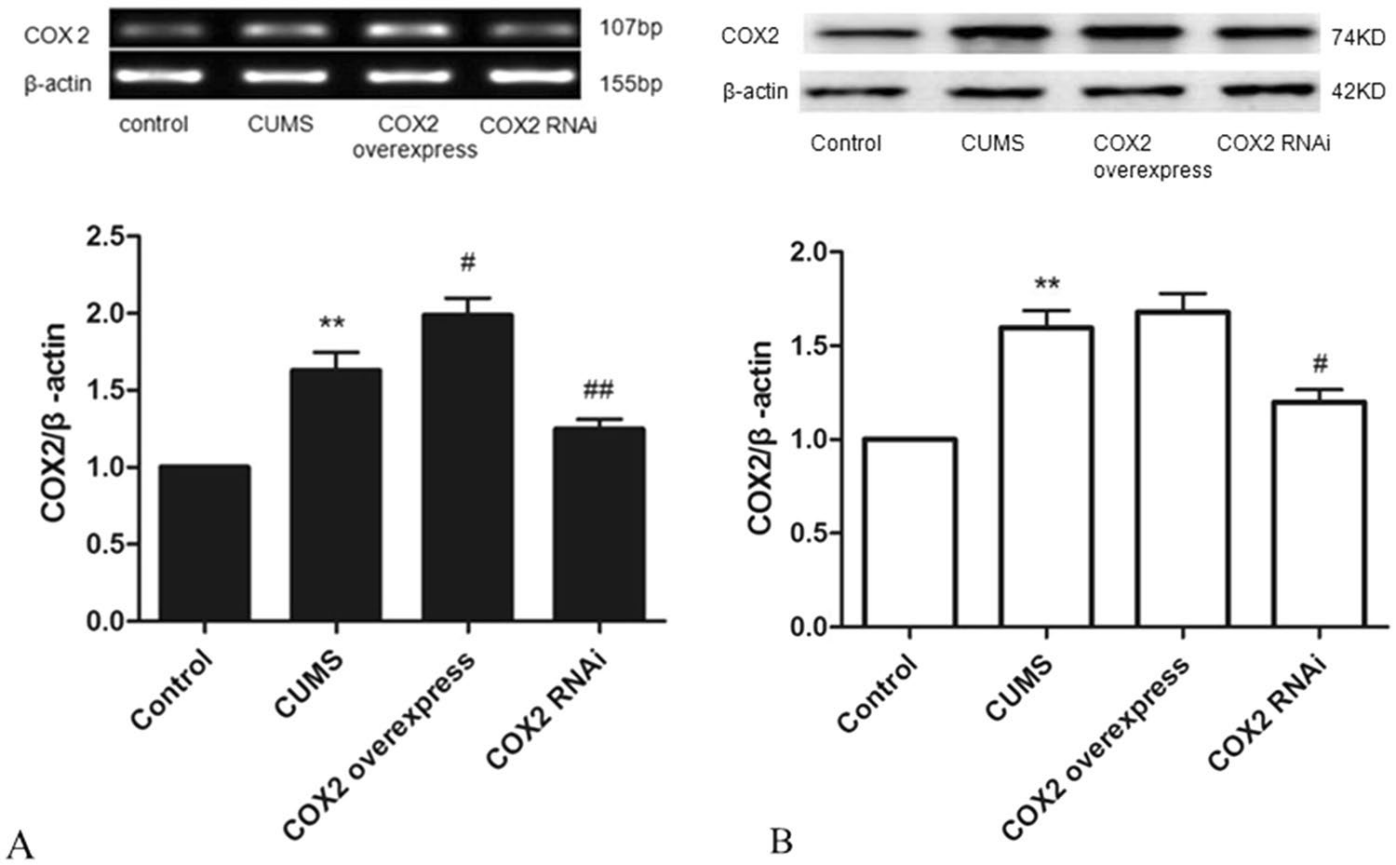
Effects of COX2 overexpression and RNAi on the expression of COX2 protein and mRNA. (**A**) Changes in COX2 mRNA expression in rat cortex. (**B**) Changes in COX2 protein expression in rat cortex. Group data shows the changes in COX2 mRNA and protein expression. Data are expressed as the mean ± SD in four separate experiments. Compared with the control group, the expression of COX2 mRNA and COX2 protein were significantly increased in the CUMS group. Compared with the CUMS group, the expressions of COX2 mRNA and protein significantly decreased in the COX2 RNAi group. However, only the expression of COX2 mRNA increased significantly in the COX2 overexpressed group. **P < 0.01 vs the control group. ^#^P < 0.05 and ^##^P < 0.01 vs the CUMS group, respectively.

The expressions of COX2 mRNA and protein in the CUMS group were significantly increased compared with those in the control group (P < 0.01). After the intraventricular injection of COX2 overexpressed lentivirus in CUMS rats, the expression of COX2 mRNA in the cortex was significantly increased (P < 0.01). However, the expressions of COX2 mRNA and protein were significantly decreased after the administration of COX2 RNAi lentivirus in CUMS rats (Fig. 7).

### Effects of COX2 overexpression and RNAi on the expressions of EP2, EP3, PKAIIα reg, BDNF and cAMP response element-binding protein (p-CREB/CREB) protein

**Figure 8:**
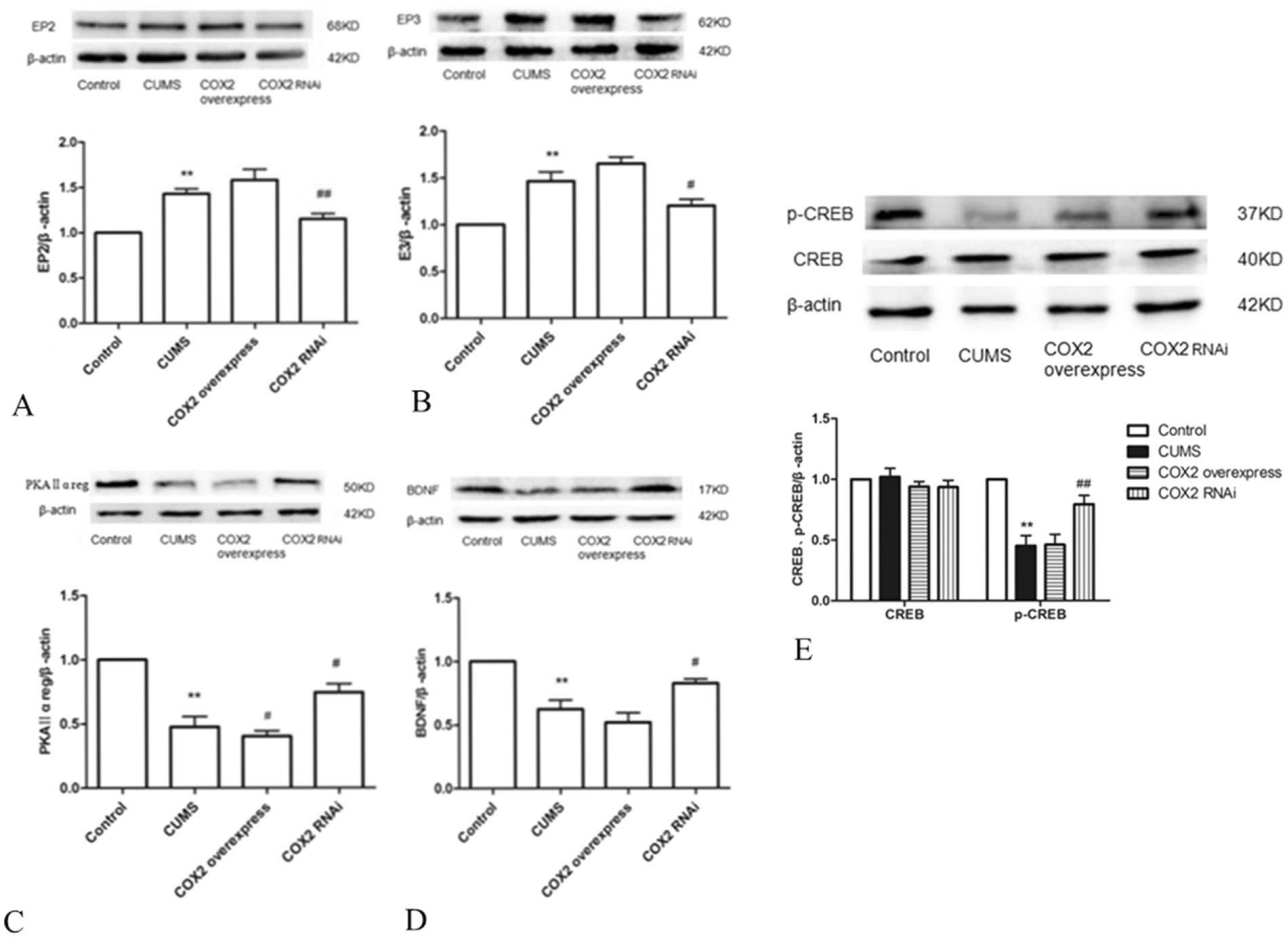
Effects of COX2 overexpression and RNAi on the expression of EP2, EP3, PKAIIα reg, BDNF and p-CREB/CREB protein. (**A**) Changes in EP2 protein expression in rat cortices. (**B**) Changes in EP3 protein expression in rat cortices. (**C**) Changes in PKAIIα reg protein expression in rat cortices. (**D**) Changes in BDNF protein expression in rat cortices. (**E**) Changes in p-CREB/CREB protein expression in rat cortices. Group data shows the changes in the expressions of EP2, EP3, PKAIIα reg, BDNF and p-CREB/CREB protein. Data are expressed as the mean ± SD from four separate experiments. Compared with the control group, the expressions of EP2 and EP3 were significantly increased in the CUMS group, while the expressions of PKAIIα reg, BDNF and p-CREB/CREB significantly decreased. Compared with the CUMS group, only the expression of PKAIIα reg significantly decreased in the COX2 overexpressed group. The expressions of EP2 and EP3 significantly decreased in the COX2 RNAi group, whereas the expressions of PKAIIα reg, BDNF and p-CREB/CREB significantly increased. **P < 0.01 vs the control group. ^#^P < 0.05 and ^##^P < 0.01 vs the CUMS group, respectively.

The levels of EP2 and EP3 in CUMS rat cortices were significantly increased compared with those in the control group, while the levels of PKAIIα reg, BDNF and p-CREB/CREB decreased significantly (P < 0.01). Compared with that in the CUMS group, the expression of PKAIIα reg decreased significantly with the administration of COX2 overexpressed lentivirus (P < 0.05), while the expressions of EP2 and EP3 significantly increased. However, changes in the p-CREB/CREB and BDNF expressions were not significant. The intraventricular injection of COX2 RNAi lentivirus significantly decreased the expressions of EP2 and EP3 and significantly increased the expressions of PKAIIα reg, p-CREB/CREB and BDNF (Fig. 8).

## Discussion

In daily life, the appropriate pressure, challenges and setbacks (stress factors) can mobilize nonspecific body reactions and increase resistance to the pathogenic factors of the outside world. However, serious, persistent, and unmanageable stress is detrimental for health^16^, especially chronic stress, which is a potential psychological burden that can not only contribute to depression but also worsen existing symptoms of depression^17, 18^. In addition, the occurrence of depression is closely related to genetic factors, individual cognition, etc. The pathogenesis of depression with complex aetiology and abnormal changes in pathophysiology is still being explored^19^.

It has been confirmed that the onset of depression is related to a variety of pro-inflammatory cytokines and inflammatory mediums^20^. Psychological stress such as fear and chronic mild stimulation can cause the release of pro-inflammatory cytokines such as IL-1β, TNF-α and IL-6 in various brain regions. COX2 and its downstream inflammatory product PGs are significantly increased in this process to trigger an inflammatory cascade reaction^21, 22^. The increasing central inflammation can further activate microglial cells to release varieties of pro-inflammatory factors. Thus, this cycle continues with cascading effects of inflammatory responses that cause abnormal depressive-like behaviour changes. Administration of COX2 selective inhibitor in depressive rats caused by olfactory bulb removal can relieve their depressive behaviours and reduce the levels of cytokines in the hypothalamus^23^. For clinical patients with serious depressive disorders, treatment with selective COX2 inhibitor can not only improve the patient’s depressive behaviour but also reduce the serum level of IL-6^24^. Thus, the hypothesis that inflammatory responses mediate depression is supported and the concentration and activity changes in COX2 have become known as key points that affect the whole process of depression.

In our present experiment, in order to explore the role of the COX2 pathway in the rat cortex in the pathogenesis of depression, we administered COX2 selective inhibitor meloxicam to inhibit the activity of COX2 enzyme and lentivirus to silence or promote COX2 gene expression. Our results indicate that CUMS caused significant effects on depressive behaviour, increased COX2 expression and the levels of TNF-α, IL-1β and MDA and decreased the SOD activity in rat cortices. Administration of COX2 inhibitor or COX2 RNAi lentivirus decreased COX2 expression and reduced the depressive behaviours in CUMS rats. Meanwhile, cortical neuronal inflammation and oxidative stress damages were relieved. The treatment of COX2 overexpressed lentivirus significantly aggravated the depressive symptoms, inflammation and oxidative stress in CUMS rats.

In the central nervous system (CNS), PGE2 is one of the optimal and most widely distributed prostaglandins produced from AA under the catalysis of COX2. It is also an important signal transmission medium for COX2 that is involved in physiological functions of the central nervous system *via* regulating changes in neurochemistry, the neuroendocrine system and behaviour. PGE2 has neurotoxic and neuroprotective effects *via* diverse specific PG receptor signalling pathways. *Hutchinson AJ* reported that endogenous PGE2 could regulate the synthesis and secretion of BDNF from human microglia and astrocytes^8^. Study results have shown that a selective COX2 inhibitor significantly reduced long-term potentiation (LTP) induction in hippocampal dentate granule neurons and that exogenous PGE2 (not PGD2 or PGF_2α_) could reverse the effects of a COX2 inhibitor^25^. These results suggest that COX2 is mainly involved in the regulation of synaptic and neural plasticity by PGE2. When the body is stimulated by diseases or various physiological and psychological external conditions, over-activation of COX2 can induce more PGE2 production beyond the natural physiological levels. When suffering from inflammation, the CNS undergoes the aberration of neuronal plasticity regulation, as well as neural structure and function at the same time.

PGE2 exerts diverse effects through binding to four different G protein-coupled receptors (EP1, EP2, EP3, and EP4), among which EP2 and EP3 show the highest expressions in the hippocampus and cortex^26^. Studies have reported that EP2 receptor agonist can imitate PGE2 to enhance the synaptic transmission and maintain neural plasticity and function^27^. Mice with the EP2 gene knocked out showed obvious cognitive disorders and anxious aggravation compared with normal mice^28^. Studies have also shown that inhibiting the expression of EP3 in the visual cortex could enhance LTP^29^. Knocking out the EP3 gene in APPSwe-PS1 ∆E9 mice can alleviate central oxidation stress and neural inflammatory injuries caused by deposition of Aβ amylum^30^. These studies together suggest that EP2 and EP3 are closely related to cognitive functioning and affective mental illness. These two receptors may be the intermediate nodes between neural plasticity changes and the activation of cortical pathway AA-COX2. Studies have demonstrated that the EP2 receptor is coupled with the G protein Gs, while the EP3 receptor is coupled with Gi. Both showed obvious biological activities by regulating the AC-cAMP signalling pathway^31^.

BDNF is widely distributed in the cerebral cortex and hippocampus, which is a main factor for neuronal plasticity regulation. It is also a necessary factor for protecting neurons against injury and promoting neuronal regeneration when suffering from damage. The expression of BDNF is closely related to the cAMP signalling pathway. As a second messenger, cAMP can activate PKA, which can catalyse the Ser^134^ site in phosphorylated CREB. The phosphorylation of CREB can also regulate the transcription of genes, such as BDNF, TrkB, c-fos, and Bcl-2, to affect cell proliferation, differentiation and apoptosis^32^. The autopsy results of depressive patients have shown that the activities of cAMP and PKA in the prefrontal cortex were decreased, especially in those who suffered from severe depression or suicide. Furthermore, the expressions of CREB and BDNF, which are regulated by CREB, were significantly lower than those in the normal group. After antidepressant treatment, the cAMP/PKA pathway was activated and the level of BDNF was negatively correlated with the depression scale scores of patients^33–35^.

Our experimental results showed that the EP2 and EP3 expressions significantly increased and that the levels of cAMP, PKA, and BDNF and the ratio of phosphorylation CREB protein and CREB protein in rat cortical neurons significantly decreased in the CUMS model group. The administration of a COX2 inhibitor and COX2 RNAi lentivirus could significantly blunt the changes in COX2, EP2, EP3, cAMP, PKA, CREB and BDNF in CUMS rat cortices. On the other hand, treatment with COX2 overexpressed lentivirus increased the EP2 and EP3 expressions and inhibited the activation of cAMP/PKA-CREB-BDNF in CUMS rat cortices.

The PGE2-EP2/EP3 signalling pathway also plays a vital role in a variety of brain injuries and neurodegenerative diseases, but the mechanism is complex and remains unknown. In a model of focal cerebral ischaemia injury, the activation of EP2 receptors can protect the brain injury caused by activated toxicity, which is a possible mechanism involved in raising cAMP signal transduction pathways^36^. However, in research on Alzheimer’s disease, knocking out the EP2 gene can reduce Aβ deposits and the injuries caused by oxidative stress^37^. These results demonstrated that based on the stimulation of different neural injury factors activation of PGE2-EP2 can produce different neural protection and/or toxic effects. At present, studies on PGE2-EPs about depression are quite few, and they have simply considered the regulation of cAMP by the activation of EP2 receptors. However, the EP3 receptors have also been shown to be activated at the same time. EP3 activation could decrease the level of cAMP, and this might offset the neural protective effects of EP2.

Our experimental results together with these existing reports indicate that the occurrence of depression is closely related to the activation of the cortical COX2 pathway. CUMS stimulation induced oxidative stress, inflammation and COX2 overexpression. The latter further activated the PGE2-EP2/EP3 signalling pathway and decreased the levels of cAMP/PKA/CREB/BDNF. As a result, cortical neuronal plasticity, neuronal structure and neuronal function were impaired, and depressive symptoms occurred. However, it is necessary to study the effect of intervention on EP2, EP3 and the related nodes of the cAMP/PKA/CREB/BDNF signalling pathway separately and/or in combination to explore the much deeper relationship between the inflammatory response caused by COX2 pathway activation and neuronal plasticity.

## Materials and Methods

### Animals

Seventy clean Sprague-Dawley male rats, weighing 180–220 g (*obtained from the animal laboratory centre of Chongqing Medical University*) were selected. Rats were kept according to the national standard set forth by the “Laboratory Animal-Requirements of Environment and Housing Facilities” (GB 14925-2001). The experiments were approved by the Animal Laboratory Administrative Center and the Institutional Ethics Committee of Chongqing Medical University (License number: SCXK YU 2012-0001) and were also in accordance with Chongqing Administration Rule of Laboratory Animals and the National Institutes of Health Guidelines.

The standard experimental conditions were as follows: temperature of 22 ± 2 °C, humidity of 50 ± 10%, alternating exposure to light and dark for 12 h, normal diet, and drinking water for 7 days to fit the experimental environment.

### Chemicals

Meloxicam (*Kunshan Rotam Reddy Pharmaceutical Co*., *Ltd*, *China*) was prepared to make suspensions of 1.0 and 3.0 mg·kg^−1^ with 0.5% sodium carboxymethylcellulose (CMC-Na) (*National Chemical Reagent*, *China*). Efficiency of COX2 RNAi, COX overexpressed lentivirus and no-load virus (*Genomeditech*, *Shanghai*, *China*) transfection and overexpression/interference were determined before the experiments.

### Establishment of animal models

After 7 days of acclimatization, rats in the CUMS group were kept separately, and methods described in the literature 11, 16 were used and improved for CUMS stimulation, including the following: clipping tail for 1 min, 4 °C cold water swimming for 5 min, 45 °C hot water swimming for 5 min, fasting from food and water for 24 h, fasting from food and water for 24 h while tilting at a 45° angle for 24 h, damp bedding for 24 h, noise stimulation (92 db, 92 Hz) for 2 h/day and alternating exposure to light and dark. A total of eight types of stimulation were performed, and the stimulation was randomly assigned to rats for daily treatment. Each treatment was performed for no more than 3 days.

### Protocols

The present experiment consisted of two parts. The first part aimed to set up the rat model of depression with CUMS and to observe the changes in behaviour and COX2 mRNA and protein expressions in rats subjected to meloxicam. In the second part, the COX2 inhibitors, COX2 RNAi- and COX2 overexpressed-lentivirus were transduced to rats to illustrate the changes in COX2 and its downstream PGE2-cAMP/PKA-CREB-BDNF pathway in depressive rat cortices.

One hundred and fifty rats that scored 40–100 in the open-field test were selected and randomly divided into two groups: normal group (n = 20) and CUMS-treated group (n = 130). Rats in the normal group were kept in large cages, with 5 rats per cage, and free water and food were provided without any stimulation, while rats in the CUMS-treated group were kept separately and given 42 days of CUMS stimulation. After that, the sucrose preference was used to evaluate the depressive behaviour, and the rats with significant depressive behaviour were used in the experimental protocol below. The rats with no significant depressive behaviours were not enrolled in the following test.

Section one: Ten rats in the normal group and 40 rats with depression were selected and were randomly divided into 4 groups. There were 5 groups: normal control group (NG), model group (MG), positive drug group (SG), meloxicam 1 mg·kg^−1^ group (MDG) and meloxicam 3 mg·kg^−1^ group (MGG). The basis of the drug dose settings for meloxicam and sertraline was in accordance with the results of our previous study^11^. Sertraline (5 mg·kg^−1^) and meloxicam (1 and 3 mg·kg^−1^) were administered to the positive drug group, and the meloxicam 1 mg·kg^−1^ and meloxicam 3 mg·kg^−1^ groups, respectively, and isovolumetric CMC-Na was given to NG and MG once per day for 21 days, po. The behavioural tests were performed after the drug intervention.

Section two: Ten rats in the normal group and thirty rats with depression were used and were divided into 4 groups: no-load virus (control) group (n = 10), no-load virus + CUMS group (n = 10), COX2 overexpressed lentivirus + CUMS group (n = 10), and CUMS + COX2 RNAi group (n = 10). Rats were intraventricularly injected with 15 μl of no-load virus, COX2 overexpressed lentivirus and COX2 RNAi lentivirus on d5, d12 and d19, respectively, after treatment of CUMS. The behavioural tests were performed 2 days after the 3rd injection test.

### Behaviour examination

The sucrose preference test, open-field test and forced swimming test were used to evaluate the depressive-like behaviours.

#### Sucrose preference test

Rats were placed in a single cage before the test and were trained to adapt to the sugary drink. During the first 24 h, each rat was given 2 bottles of 1% sugar water, and during the following 24 h, each rat was given 1 bottle of water and 1 bottle of 1% sugar water. The sequence of bottles was then changed every 2 h. After the training, 24 h of fasting treatment was performed. Then, each rat was given 1 bottle of water and 1 bottle of 1% sugar water, which were weighed in advance. The consumption of each bottle was measured 1 h later. The sugar water preference rate of the rats was calculated according to the formula of water consumption/(sugar water consumption + water consumption) × 100%.

#### Open-field test

The test was conducted in a dark and quiet room with 5 m visibility distance. A case was used as the experimental device, which was painted black on the bottom and had no covering, with a length of 100 cm, width of 50 cm, and height of 100 cm. The bottom of the case was marked with white lines that created white squares of 20 cm × 20 cm. At the beginning of the test, the rat was placed in the central square following activities were observed for 300 s: the number of squares passed, which were recorded as the horizontal movement, with all feet in the same grid, which was defined as one movement; and the total number of instances where the rat was up-right on its hind legs, indicating the rising of its two front paws or the number of times walls were climbed, which were recorded as the vertical movement.

#### Forced swimming test

For each test, a rat was put into a transparent cylindrical bucket with a diameter of 30 cm, height of 50 cm, water depth of 30 cm, and water temperature of (24 ± 2) °C. After 2 min of swimming, the test started on the 3^rd^ min. The duration of stationary state in 300 s of each rat was recorded. Stationary state refers to when rats floated on the water without struggling or only the head of rat floated on the water with few body activities.

### Immunohistochemical Staining

Immunohistochemistry was performed to investigate the expression of BDNF in rat cortices. After finishing all the behaviour function tests, 4 rats of each group were intraperitoneally anesthetised with 4% chloral hydrate (10 ml·kg^−1^) and transcardially perfused with saline (100 ml) followed by 4% paraformaldehyde in phosphate-buffered saline (200 ml). The rat brain was removed and stored in the same fixative solution. The cortex of the brain was isolated and sliced into 5-μm-thick sections. Briefly, cortex sections of 4 rats from each group were dewaxed and rehydrated in ethanol with decreased concentration. The sections were then blocked in methanol for 20 min at room temperature for endogenous peroxidase in 3% H_2_O_2_. Slides were washed with PBS three times (5 min for each time) and were pre-incubated in 1% serum for 30 min at room temperature. Thereafter, the sections were incubated with primary antibodies BDNF (dilution 1:50, *Santa*, *USA*) overnight at 4 °C. The sections were incubated with biotinylated secondary antibody (dilution 1:100) for 30 min at 37 °C followed by incubation with streptavidin for 20 min. Then, the sections were washed with PBS another 3 times (5 min each) before the reaction with DAB solution. The sections were counterstained with haematoxylin and then observed under light microscopy.

### Measurement of MDA content and SOD activity

On the second day after completion of the behaviour function test, the rats in each group (n = 6) were anesthetised and the cerebral cortices were separated. The SOD activity and MDA content were detected according to the instruction manual of the kit (*Jiancheng Bioengineering Ltd*, *Nanjing*, *China*). The protein content was measured using the BCA protein assay kit (*Beyotime*, *China*).

### Enzyme-Linked Immunosorbent Assay (ELISA)

The rat cortices of each group were removed on the second day after completion of the behaviour function tests (n = 6). ELISA kits were used to detect the levels of PGE2 (*TaKaRa*, *Japan*), cAMP (*R&D Systems China*, S*hanghai*, *China*), TNF-α (*HuaMei Bioengineering Ltd*, *Wuhan*, *China*) and IL-1β (*HuaMei Bioengineering Ltd*, *Wuhan*, *China*).

### Western blot Test

40 mg of rat cortex (n = 4) was added to 0.4 ml of tissue lysate solution for protein extraction and was centrifuged at 12,000 × g for 15 min at 4 °C. The supernatant was then used for western blotting. The protein concentrations were determined with the BCA protein assay kit (*Beyotime*, *China*). A 10 μl sample of each protein was separated by sodium dodecyl sulphate polyacrylamide gel electrophoresis (SDS-PAGE) and transferred to polyvinylidene fluoride (PVDF) membranes (*Millipore*, *USA*). The membranes were blocked with 5% BSA for 1 h at room temperature and then were probed with specific primary antibodies, including COX2, anti-EP_1_, EP_2_, p-CREB/CREB, BDNF, PKAIIα reg (dilution 1:500; *Santa*, *USA*) and β-actin (dilution 1:3000; *Proteintech*, *USA*) overnight at 4 °C. The membranes were washed three times in TBST and were incubated with HRP-conjugated secondary antibodies at room temperature for one hour. After three washes in TBST, protein signals were visualized by ECL (*Bio-Rad*, *USA*).

### RT-PCR Test

To determine the expression of COX2 mRNA in rat cortices, total RNA was extracted from the cortex using RNAiso Plus reagent (*Takara*, *Japan*). cDNA templates were generated by reverse transcription kit (*Takara*, *Japan*) following the manufacturer’s instructions and were amplified using the MIX PCR kit (*Takara*, *Japan*). PCR products were separated by 2% agarose gel electrophoresis and visualized by ethidium bromide staining. All the samples were normalized by the expression level of β-actin. The absorbance values of COX2 and β-actin mRNA were measured with a Bio-Rad gel imaging analysis system (*Bio-Rad*, *USA*). The primer sequences for COX2 were: forward 5′-TGAACACGGACTTGCTCACTTTG-3′ and reverse 5′-AGGCCTTTGCCACTGCTTGTA-3′ (107 bp), β-actin with forward 5′-ACGGTCAGGTCATCACTATCG-3′ and reverse 5′-GGCATAGAGGTCTTTACGGATG-3′ (155 bp).

### Statistical analysis

The results were expressed as the means ± standard deviation (SD) and analysed with the use of SPSS 12.0 (*SPSS Inc*. *Chicago*, *US*). Within-group variances were compared with the use of Dennett’s t-test. P < 0.05 and P < 0.01 were seen as statistically significant.

## Electronic supplementary material

The supplementary informations about the original figures
