# Supplementary material for: Cyclooxygenase-2 Signalling Pathway in the Cortex is Involved in the Pathophysiological Mechanisms in the Rat Model of Depression
Source: Sci Rep. 2017 Mar 28;7:488. doi: 10.1038/s41598-017-00609-7 (PMC5428817; doi:10.1038/s41598-017-00609-7)

Cyclooxygenase-2 Signalling Pathway in the Cortex is involved in the Pathophysiological Mechanisms in the Rat Model of Depression

Qi Chen[1], Ying Luo[1], Shengnan Kuang[1], Yang Yang[1], Xiaoyan Tian[1], Jie Ma[1], Shaoshan Mai[1], Lai Xue[1], Junqing Yang[1*]

1 Department of Pharmacology, Chongqing Medical University, the Key Laboratory of Biochemistry and Molecular Pharmacology, Chongqing 400016, China

[*]Corresponding author:

Prof. Junqing Yang,

Department of Pharmacology, Chongqing Medical University, Chongqing 400016, China;

Tel: +86-23-68485161; Fax: +86-23-68485161;

E-mail: 1139627371@qq.com

Qi Chen and Ying Luo are co-first authors.

Supplementary Figure 3 The Original Western- blotting figure of PKAII α reg protein for Figure 3

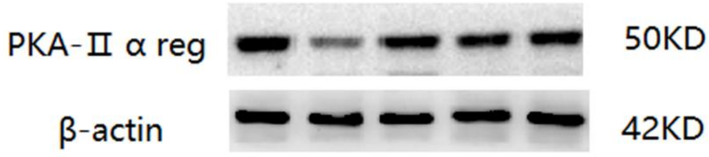

NG MG SG MDG MGG

Supplementary Figure 7 The Original Western- blotting figure of COX2 protein for Figure 7

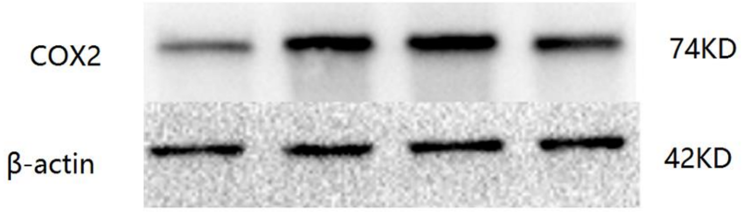

Control CUMS COX2 overexpress COX RNAi

Supplementary Figure 7 The Original RT-PCR figure of COX2 mRNA for Figure 7

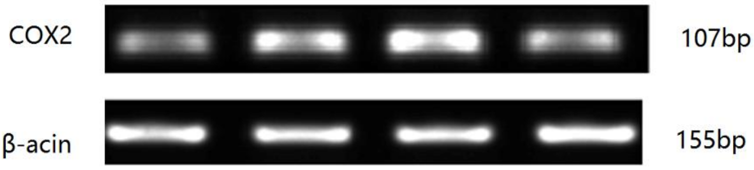

Control CUMS COX2 overexpress COX RNAi

Supplementary Figure 8 The Original Western- blotting figure of EP2 protein for Figure 8

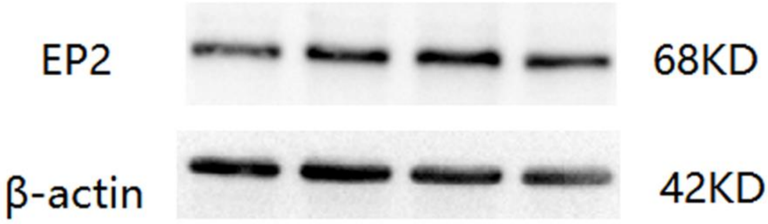

Control CUMS COX2 overexpress COX RNAi

Supplementary Figure 8 The Original Western- blotting figure of EP3 protein for Figure 8

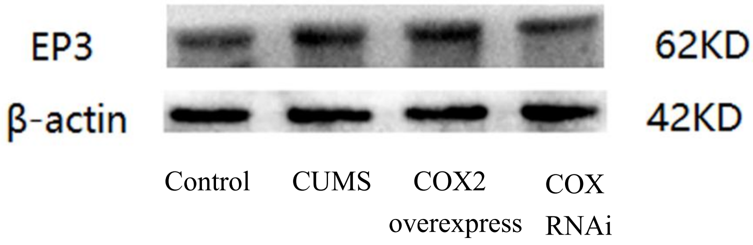

Supplementary Figure 8 The Original Western- blotting figure of BDNF protein for Figure 8

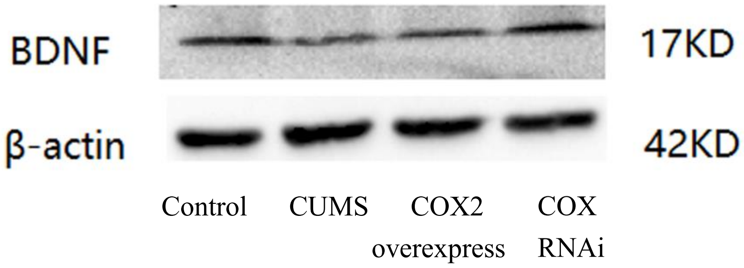

Supplementary Figure 8 The Original Western- blotting figure of PKA-II α reg protein for Figure 8

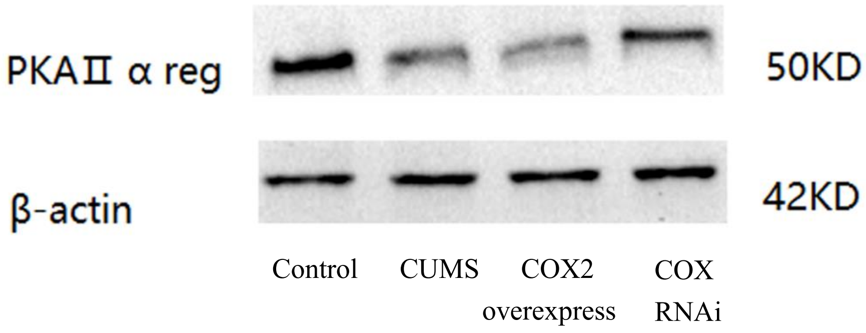

Supplementary Figure 8 The Original Western- blotting figure of p-CREB protein for Figure 8

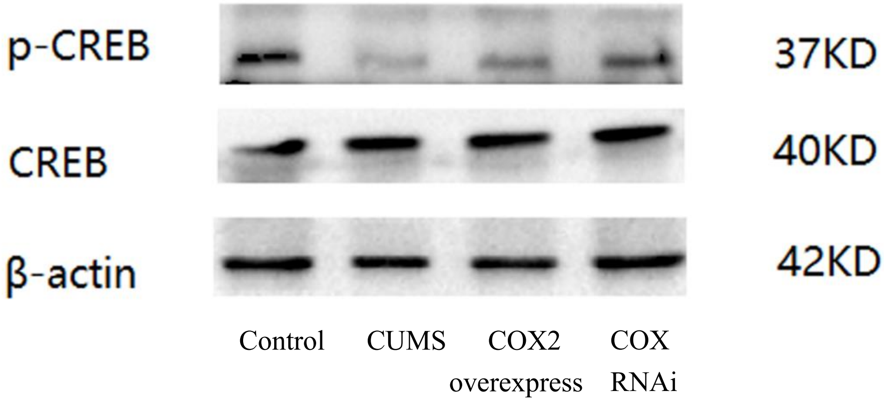

Supplement: Supplementary file 1 — The supplementary informations about the original figures [file 41598_2017_609_MOESM1_ESM.pdf]
